# Pharmacogenomic and Pharmacotranscriptomic Profiling of Childhood Acute Lymphoblastic Leukemia: Paving the Way to Personalized Treatment

**DOI:** 10.3390/genes10030191

**Published:** 2019-03-01

**Authors:** Sonja Pavlovic, Nikola Kotur, Biljana Stankovic, Branka Zukic, Vladimir Gasic, Lidija Dokmanovic

**Affiliations:** 1 Laboratory for Molecular Biomedicine, Institute of Molecular Genetics and Genetic Engineering, University of Belgrade, 11000 Belgrade, Serbia; nikola0104@gmail.com (N.K.); bi.stankovic@gmail.com (B.S.); branka.petrucev@gmail.com (B.Z.); vlada.gasic42@gmail.com (V.G.); 2 University Children’s Hospital, 11000 Belgrade, Serbia; lidija.dokmanovic@udk.bg.ac.rs; 3 Faculty of Medicine, University of Belgrade, 11000 Belgrade, Serbia

**Keywords:** pharmacogenomics, pharmacotranscriptomics, high-throughput analysis, childhood acute lymphoblastic leukemia

## Abstract

Personalized medicine is focused on research disciplines which contribute to the individualization of therapy, like pharmacogenomics and pharmacotranscriptomics. Acute lymphoblastic leukemia (ALL) is the most common malignancy of childhood. It is one of the pediatric malignancies with the highest cure rate, but still a lethal outcome due to therapy accounts for 1–3% of deaths. Further improvement of treatment protocols is needed through the implementation of pharmacogenomics and pharmacotranscriptomics. Emerging high-throughput technologies, including microarrays and next-generation sequencing, have provided an enormous amount of molecular data with the potential to be implemented in childhood ALL treatment protocols. In the current review, we summarized the contribution of these novel technologies to the pharmacogenomics and pharmacotranscriptomics of childhood ALL. We have presented data on molecular markers responsible for the efficacy, side effects, and toxicity of the drugs commonly used for childhood ALL treatment, i.e., glucocorticoids, vincristine, asparaginase, anthracyclines, thiopurines, and methotrexate. Big data was generated using high-throughput technologies, but their implementation in clinical practice is poor. Research efforts should be focused on data analysis and designing prediction models using machine learning algorithms. Bioinformatics tools and the implementation of artificial i Lack of association of the CEP72 rs924607 TT genotype with intelligence are expected to open the door wide for personalized medicine in the clinical practice of childhood ALL.

## 1. Introduction

Emerging high-throughput technologies, which enable the analysis of individual genomes, epigenomes, transcriptomes, proteomes, metabolomes, and microbiomes, so called “omics”, have brought great advancements in the field of biomedical sciences [1]. Moreover, multiple genomic, epigenomic, transcriptomic, and proteomic markers have already been included in routine diagnostic, prognostic, and therapeutic protocols for a great number of diseases [2,3]. This is important for designing new therapies, like molecular and gene therapy, which is the basis of personalized medicine.

Pharmacogenetics and pharmacogenomics are staples of personalized medicine. The goal of pharmacogenomics is to create an effective therapy strategy based on the genomic profile of a patient. Pharmacotranscriptomics is a field of study which investigates associations between variations in the transcriptome with the pharmacokinetics and the pharmacodynamics of drugs to detect interindividual differences between patients, so that a more efficient dose regimen of a drug can be established.

There are two main approaches in pharmacogenomics and pharmacotranscriptomics: One based on candidate pharmacogenes/pharmacotranscripts, the other based on testing the entire genome/transcriptome (genome-wide association studies/transcriptome-wide association studies (GWAS/TWAS)) for pharmacogenomic/pharmacotranscriptomic markers. Candidate genes/transcripts studies have high statistical power, but their weakness is the fact that they lack the capacity to discover new genes or transcripts. On the contrary, the strength of GWAS/TWAS lies in the ability to identify relevant pharmacogenomic or pharmacotranscriptomic markers regardless of whether their function was previously known, but they have low statistical power due to the number of independent tests performed [4]. 

Acute lymphoblastic leukemia (ALL) is the most common malignancy of childhood, accounting for around 30% of all childhood cancers and around 80% of all childhood leukemia. It is one of the pediatric malignancies with the highest cure rate [5]. However, more than 10% of patients experience an unfavorable outcome.

Considering that more efficient treatment of pediatric ALL has not been achieved by the introduction of novel drugs into the treatment protocols, but instead by trying to diminish the adverse effects of the drugs that are already included in the protocols, it is understandable that pharmacogenomics and pharmacotranscriptomics have become very important in this field. 

In the current review, we present the results of pharmacogenomics and pharmacotranscriptomics studies conducted in pediatric ALL using high-throughput technologies. We aim to summarize the contributions of these novel technologies in this field to find out what additional opportunities they offer and to suggest future directions.

## 2. Childhood Acute Lymphoblastic Leukemia

Acute lymphoblastic leukemia (ALL) is a rare disease, representing about one fourth of all cancers in children. The incidence rate of ALL among children aged up to 14 years is about 41:1,000,000, with a peak in children aged 2–7 years. Biologically, the disease originates from T- and B-lymphoid precursors of the bone marrow [6].

In childhood ALL, almost all patients achieve remission, and about 85% of the patients are expected to be cured with modern treatment protocols [7]. The treatment of the patients with ALL is usually tailored according to risk group stratification defined by clinical and laboratory features [8]. Standard treatment options for childhood ALL encompass historically validated cytotoxic agents grouped into so called therapeutic phases or elements. These include remission induction chemotherapy agents—vincristine, a corticosteroid drug, anthracyclines, and asparaginase [9]. Post-induction (or consolidation) treatment options for childhood ALL include cyclophosphamide, cytarabine, 6-mercaptopurine, and high-dose methotrexate. Most protocols also include an intensification phase, utilizing the same drugs, namely vincristine, corticosteroids, anthracyclines, and cytarabine, combined with another thiopurine, such as 6-thioguanine [10]. After completing intensive treatment phases, the patient is due for maintenance therapy, whose backbone is based on daily oral 6-mercaptopurine and weekly oral methotrexate.

Hematopoietic stem cell transplantation also has a role in the treatment of ALL patients, such as those with unfavorable clinical and laboratory features as well as patients with relapsed disease.

Efficient therapy causes side effects in 75% of childhood ALL patients [11]. Aside from this, chemotherapy leads to delayed side effects and even permanent sequelae [12,13]. It is estimated that 1–3% of pediatric ALL patients have a lethal outcome due to the consequences of treatment side effects and not due to the consequence of the disease [8,14].

It is necessary to emphasize that a patient with a malignancy has two genomes: The constitutional genome, characteristic for all cells except the tumor clone; the other is the tumor genome that contains acquired genetic variants and which changes during the evolution of tumor clones. Variants in the constitutional genome and germinative variants influence the transport and the metabolism of drugs, making them responsible for the efficacy of the drugs and the side effects, while somatic mutations are responsible for the resistance of the tumor to drugs [15].

The side effects of therapy in pediatric ALL are a consequence of the insufficient specificity of drugs, the small therapeutic index of drugs, and the high exposure and long-term application of drugs. The most frequent complications are hypersensitive reactions, neuro-, cardio-, and hepatotoxicity, the toxicity of the digestive tract and kidneys, as well as myelosuppression and osteonecrosis [11]. General toxicity can be diminished by patient stratification, while individual patient toxicity caused by genetic variants of the genes responsible for drug metabolism can be prevented with specific genetic tests and individually tailored chemotherapy [16,17].

## 3. Glucocorticoid Drugs

Synthetics glucocorticoids (GCs) are some of the most frequently used drugs in the treatment of immune or inflammatory diseases, like inflammatory bowel disease, asthma, allergic rhinitis, etc. The capability of GCs to induce apoptosis in thymocytes, monocytes, and peripheral T cells makes them a central component in chemotherapeutic protocols in the treatment of leukemia, lymphomas, and myelomas. GCs drugs, prednisone and dexamethasone, represent the basis of chemotherapy in childhood ALL. The cytotoxic effect of GCs is connected with their antiproliferative effect, which is realized in specific cell types using the glucocorticoid receptor (GR) [18]. A proposed mechanism of action of GCs in lymphoblasts is that they activate the Bim protein, which induces apoptosis and deactivates NF-κB and AP1, thus leading to a negative modulation in cell survival [19]. The most important side effects of GCs drugs are osteonecrosis, sepsis, diabetes, myopathy, hypertension, and behavioral disorders.

The mechanisms of the GCs response in childhood ALL are not well-known yet. Despite confusing results of candidate genes studies, some variants in pharmacogenes could be considered as possible pharmacogenomic markers of the GCs response in ALL.

One of the most important pharmacogenes is the *NR3C1* gene that encodes the GR. Most frequently studied variants in this pharmacogene, like rs6189/rs6190 (ER22/23EK) and rs56149945 (N363S), have not shown a significant association with the therapeutic response to GCs in ALL [20,21]. Another extensively studied variant, rs41423247 (*BclI* variant), has shown an association with the therapeutic response [22].

It has been shown that the presence of the minor allele of variant rs6198 in the 3’UTR region of the *NR3C1* gene is associated with a poor response to GCs in pediatric ALL [23]. The variants, rs33389 and rs33388, have shown to affect the GCs response only when they act as a haplotype [23,24]. On the other hand, the rs33389 C allele and rs33388 T allele as a part of *NR3C1* ACT haplotype (rs41423247-rs33389-rs33388) are strongly associated with GCs sensitivity.

*ABCB1* is another pharmacogene relevant to the GCs response that has deserved special attention in candidate gene studies. It encodes for a membrane transporter, P-glycoprotein, an efflux transporter that ejects xenobiotics. The haplotype, *ABCB1* CGT (rs1128503-rs2032582-rs1045641), was found to be associated with a poor GCs response and increased the risk of relapse in the induction remission phase of childhood ALL therapy [25,26]. Until now, only the *ABCB1* C3435T variant was associated with adverse effects, i.e., bone marrow toxicity [25] and grade 1 and grade 2 infections [27].

Glutathione S-transferases (*GSTs*) are genes of the same gene family that encode detoxification enzymes, which initiate the process of elimination of xenobiotics. Three enzymes of this enzyme family have been studied extensively in the context of the GCs response in childhood ALL: GSTM1, GSTT1, and GSTP1. When it came to *GSTT1*, conflicting results were reported [23,28,29,30]. Variants in *GSTM1* were shown to be associated with the severity of infections [27] and an increased risk of relapse [30]. In one study, it was shown that the *GSTP1* GCs (rs1695-rs1138272) haplotype was associated with a good response to GCs in the remission induction phase of childhood ALL therapy [23].

A variant, rs1876829, in the *CRHR1* gene was shown to be associated with GCs-induced hypertension in childhood ALL [31]. While there are other candidate genes (*ST13, STIP1, FKBP5*) whose products participate in the GCs pathway, they have not been studied in the context of the GCs response in childhood ALL. Generally, there are not many pharmacogenomics studies related to the GCs response in childhood ALL using the candidate gene approach.

As for candidate transcripts studies, they are even fewer. A higher expression of *ABCB1*, related to the *ABCB1* CGT (rs1128503-rs2032582-rs1045641) haplotype, was found to be associated with GCs resistance [23].

Unlike the candidate gene approach, using high throughput methods in association studies of the GCs response could point to relevant variants or clusters of variants that could be quite important in determining the differences in the GCs response between childhood ALL patients. Novel pharmacogene variants in pediatric ALL could be essential as prognostic and/or predictive biomarkers for selecting the best dose and the right time for GCs treatment of this malignancy [32].

In one GWAS study [33], 440,044 single nucleotide polymorphisms (SNPs) which contributed to the risk of relapse were studied in 2535 childhood ALL patients, after adjusting the studied cohort of patients for genetic ancestry and therapeutic regimens. Of the 134 newly found SNPs associated with the risk of relapse, four SNPs (rs6007758, rs41488548, rs10264856, rs4728709) were found to be associated with a higher clearance of dexamethasone, two of which (rs10264856, rs4728709) were located in the *ABCB1* gene, which was also considered as a pharmacogene for GCs therapy using the candidate gene approach.

In another GWAS [34], it was found that the single region of chromosome 14, which contains *SERPINA6/SERPINA1* genes, accounts for around 1% of the variance of plasma cortisol levels. Using an Illumina Exome chip and the meta-analysis of GWAS, one SNP, rs12589136, was found to influence the binding activity of the reactive center loop of the corticosteroid-binding globulin. This led to higher plasma cortisol levels and higher cortisol binding activity. Thus, variant rs12589136 was shown to influence plasma cortisol levels, which could be a future potential target of investigation when it comes to GCs therapy outcomes in childhood ALL patients.

The toxicity of GCs treatment is a generally acknowledged problem in the remission induction therapy of childhood ALL. Osteonecrosis due to the administration of dexamethasone for treating high-risk ALL patients is one of the most dangerous toxicity events of GCs treatment in childhood ALL. One GWAS study [35] found that the SNP, rs10989692, near the glutamate receptor gene, *GRIN3A*, was associated with osteonecrosis. This association was supported by two replication studies of independent cohorts of patients treated with GCs for various medical conditions. The SNP, rs10989692, could be a germline genetic variant that predisposes to glucocorticoid-induced osteonecrosis. In another GWAS study, SNPs in the *ACP1* gene (acid phosphatase 1) were associated with an increased risk of osteonecrosis during dexamethasone treatment of pediatric ALL [36]. The gene, *ACP1*, is important for regulating cholesterol and triglyceride levels [37], meaning that lipid levels are possibly relevant in the pathology of osteonecrosis in pediatric ALL.

Research in the field of pharmacotranscriptomic markers of the GCs response in childhood ALL is still new and insufficient. However, some results have been reported. The long noncoding RNA GAS5 was shown to be associated with a poor GCs response in childhood ALL during remission induction therapy [38]. GAS5 imitates the glucocorticoid response element (GRE) sequence, which is a DNA sequence to which the GC-GR complex needs to bind to in order to realize its effect, thus GAS5 can bind the GC-GR complex and stop it from binding to the GRE sequence [39]. Additionally, the association between two microRNAs, hsa-miR-142-3p and hsa-miR-17-5p, and GCs resistance in pediatric ALL was found using a semantics-oriented computational approach [40].

Microarray gene expression analyses have shown that the modified expression of genes coding for several proteins or transcription factors can be associated with GCs resistance in pediatric ALL. Epithelial membrane protein 1 (EMP1) expression was shown to be higher in prednisone poor responders, unlike in prednisone good responders [41]. EMP1 is a protein that promotes phosphorylation of Src and FAK [42]. The Src kinase family is essential in lymphocyte receptor signaling [43,44]. In another microarray study, the expression of caspase 1 (CASP1) and its activator, NLRP3, was shown to be increased as a result of poor methylation of their promoters. The elevated level of CASP1 results in intensive cleavage of the GR and increased GCs resistance [45].

Genes involved in chromatin remodeling represent another group that shows potential in contributing to the outcome of the GCs response in childhood ALL. One study using microarrays has shown that a decreased expression of three subunits of the SWI/SNF complex (*SMARCA4, ARID1A*, and *SMARCB1*) is associated with GR resistance [46]. Furthermore, when *CREBBP*, a gene which encodes the transcription coactivator and histone-acetyltransferase CREB-binding protein, was investigated using sequencing analysis, later confirmed with gene expression arrays, it was found that damaging mutations in the *CREBBP* gene contributed to GCs resistance [12].

## 4. Vincristine

The vinca alkaloid vincristine (VCR) is widely used as an anticancer drug in both solid tumors and other malignancies. VCR’s cytotoxic effects are achieved by the disruption of the mitotic spindle microtubules as VCR binds to tubulin dimers. In this way, mitotic arrest is induced and leukemic cells die during metaphase [47]. The toxicity of VCR consists of a peripheral neuropathy described by neuropathic pain and sensory and motor dysfunction, causing the necessary decrease of the VCR dose, the discontinuation of the ALL treatment, and morbidity.

A number of candidate genes involved in different aspects of VCR metabolism have been assessed for an association with both sensory and motor peripheral neuropathies related to VCR treatment in pediatric ALL patients. However, this kind of study has produced inconsistent data on genetic variants associated with an increased risk of VCR-related neuropathy and also on their significance [13,48,49,50,51,52,53,54,55]. Nevertheless, evidence from multiple studies demonstrated that the CYP3A family of enzymes is responsible for the metabolism of the VCR. The most important among them is the CYP3A5 enzyme, and variations in the *CYP3A5* gene could be essential for VCR-related side effects in pediatric patients with ALL [49,56]. Namely, the most VCR-toxicity related *CYP3A5* allelic variant in Caucasians includes CYP3A5*3, with a single nucleotide change in intron 3 leading to a premature termination codon. Patients that are carriers of the CYP3A5*3/*3 genotype with essentially no *CYP3A5* expression have severe VCR-related neurotoxicity side effects [57].

An agnostic approach was applied in GWAS, and the results showed that an inherited variant in the promoter region of the *CEP72* gene (rs924607, risk genotype TT) was associated with a higher prevalence and severity of VCR-related peripheral neuropathy in children with ALL, during the two years of continuation therapy [58]. Homozygous carriers of the *CEP72* rs924607 risk TT genotype had a cumulative risk of neuropathy that was significantly higher and the mean severity of neuropathy was significantly greater compared with all other patients. *CEP72* encodes a centrosomal protein indispensable for microtubule formation. This genomic variant generates a binding site for a NKX-6.3 transcriptional repressor in the *CEP72* gene promoter and consequently affects the decrease of *CEP72* mRNA expression, endangering microtubule stability. The same study employed shRNA impairment of the *CEP72* mRNA expression in in vitro model systems and confirmed findings that reduced *CEP72* expression in induced pluripotent stem neuronal cells as well as in leukemia cells increases their sensitivity to VCR. The same findings were confirmed using primary ALL cells from ALL patients who were homozygous carriers of the *CEP72* rs924607 TT risk genotype.

The retrospective replication study showed no association between VCR-related neurotoxicity during the induction phase of the ALL treatment and the *CEP72* rs924607 risk TT genotype [59]. The distinctive genetic background of the two analyzed populations and/or possible mechanisms causing peripheral neurotoxicity in the early or late phases of ALL treatment could be the reason. Also, the precise number of VCR doses and the overall length of VCR treatment should be considered when assessing VCR-related neurotoxicity [60]. It is possible that other genetic markers in *CEP72* or other genes (like *CYP3A5*) [61] were not taken into account when the replication study was done. It is important to notice that the end points’ or “phenotypes’” precise definitions are equally vital to understand when assessing the pharmacogenetics potential of a given marker, as well as the sole genetic variations associated with the phenotype [62].

A study using targeted sequencing and RNA-sequencing revealed that genetic variants in the VCR transporter gene, *ABCC2* (rs3740066 GG and rs12826 GG risk genotypes), were associated with VCR-related neurotoxicity during the induction phase in pediatric ALL patients [63]. Furthermore, a statistically significant protective haplotype, formed by rs3740066–rs3740065–rs12826–rs12762549–rs11190298 (ATAGG) in the *ABCC2* gene, was identified.

Recently, a whole-exome sequencing analysis combined with an exome-wide association study was performed to find out genetic risk factors for VCR-related neurotoxicity [64]. The study identified two variants significantly associated with an increased risk of high-grade VCR-related neurotoxicity, rs2781377 in the *SYNE2* gene and rs10513762 in the *MRPL47* gene. Additionally, variant rs3803357 in the *BAHD1* gene played a protective role regarding neurotoxicity. The *SYNE2* gene or *Nesprin*-2 codes for a protein with an important role in various cellular and nuclear functions [65]. The *MRPL47* gene codes for the mitochondrial ribosomal proteins involved in the oxidative phosphorylation system and, through reduced adenosine triphosphate (ATP) production, the variants in this gene could affect neuropathies, myopathies, and developmental disorders [66]. The *BAHD1* gene, an important regulator of gene silencing, already associated with tumor suppression and inflammation [67], could be connected to sensory and autonomic neuropathy via an epigenetic mechanism [68].

A recent GWAS identified genetic variants, rs1045644 in the coagulation factor C homology (*COCH*) gene and rs7963521 associated with the regulation of chemerin plasma levels, as being significantly associated with VCR-related neuropathy in ALL children [69]. Variant rs1045644 in the *COCH* gene has already been associated with progressive hearing loss and vestibular imbalance [70]. Variant rs7963521, acting through chemerin protein, influences the chemokine like receptor 1, G protein-coupled receptor 1, and the C-C chemokine-like receptor 2, thus affecting various processes, including angiogenesis, adipogenesis, osteoblastogenesis, diabetes, and inflammatory reactions [71]. The involvement of the *CEP72* gene previously reported in VCR-related toxicity [58] was not confirmed in this study [69].

An initial microRNA expression study pointed out involvement of miR-125b, miR-99a, and miR-100 in resistance to VCR and daunorubicine treatment in different major subtypes of pediatric acute leukemia [72]. MiR-125b was expressed significantly higher in patients resistant to VCR or daunorubicine, specifically in *ETV6-RUNX1*-positive ALL patients. Both miR-99a and miR-100 showed an increased expression in ALL children with VCR and daunorubicine resistance, similar to miR-125b. MiR-125b, miR-99a, and miR-100 are co-expressed in acute pediatric ALL [72]. Interestingly, the individual overexpression of these miRNAs did not induce VCR resistance, but miR-125b in combination with miR-99a or miR-100 induced a significant resistance to VCR, resulting in the concept of the synergistic drug resistance modifying effect of combined miRNAs expression [73]. Eleven genes, including four genes encoding ribosomal proteins, were significantly downregulated in *ETV6-RUNX1*-positive cells expressing high levels of miR-125b together with miR-100 and/or miR-99a [73].

A microarray analysis was used in the study, which revealed the association of the rs12894467 risk allele T with the premature mir-300 and toxicity in the induction phase of ALL treatment [74]. In fact, the rs12894467 risk allele T leads to an upregulation of miR-300, whose target among others are the transporters, ABCB1 and ABCC1, involved in VCR detoxification. An association between rs639174 in *DROSHA* and vomiting was also found.

A recent high-throughput study [75] identified the A allele of rs12402181 in the seed region of miR-3117-3p that could increase the efflux of the VCR through the *ABCC1* and *RALBP1* gene, and C allele of rs7896283 in a pre-mature sequence of miR-4481, which could be involved in the regulation of the axon guidance pathway genes and peripheral nerve regeneration, processes that are significantly associated with VCR-related neurotoxicity. The *ABCC1* gene codes for the multidrug resistance protein 1, which mediates the efflux of a broad range of antineoplastic drugs, including VCR and variants that alter the transporter functions and have already been associated with VCR-related neurotoxicity [76].

## 5. Asparaginase

Asparaginase is an enzyme that catalyzes the hydrolysis of the amino acid, asparagine (Asn), into aspartic acid (Asp) and ammonia. In general, leukemic cells do not synthesize Asn like normal cells, and are therefore dependent on its exogenous input [77]. The introduction of asparaginase leads to a circulating Asn deficit, depriving the leukemic cell of exogenous Asn, and resulting in leukemic cell death.

An asparaginase enzyme comes from various bacterial sources. However, only *Escherichia coli* and *Erwinia chrysanthemi* asparaginase are used in medicine. *Erwinia* asparaginase has been found to have less toxicity, but also less efficacy than native *E. coli* asparaginase [78]. Polyethylene glycol (PEG) asparaginase, native *E. coli* asparaginase covalently linked to PEG, decreases proteolysis, increases the drug’s half-life, and decreases the immunogenicity of the native *E. coli* asparaginase with a corresponding efficacy [79].

Toxicities, like hypersensitivity, pancreatitis, coagulation abnormalities, encephalopathy, and liver dysfunction, were reported to be related to asparaginase treatment. In cases of serious adverse drug reactions, asparaginase therapy may be altered or withdrawn in some patients.

Early candidate gene approach studies identified certain genetic variants associated with adverse drug reactions in children with ALL that received asparaginase during standard treatment ALL protocol. An analysis of the genes coding for proteins in the asparaginase pathway (asparagine synthetase—ASNS, the basic region leucine zipper activating transcription factor 5—ATF5, and arginosuccinate synthase 1—ASS1) identified the genetic variation in the *ATF5* gene, T1562C, that affects the activation of endogenous asparaginase transcription after nutrition deprivation, influencing ATF function and responses to treatment in ALL children [80]. Further study of asparaginase action pathway genes revealed that the 3R3R *ASNS* genotype was correlated with pancreatitis and allergies in ALL patients [81].

A “hypothesis-free” exome-wide association study (EWAS) was performed on whole exome sequencing (WES) data [82], indicating that the rs3809849 in the *MYBBP1A* gene was associated with an allergy, pancreatitis, and thrombosis related to asparaginase use. The same genetic variant was also associated with a reduction in event free survival and overall survival. The *MYBBP1A* gene encodes the MYB binding protein 1a, involved in many essential cellular processes, including cell cycle control, mitosis, the nuclear stress response, and tumor suppression [83]. This protein is also a co-repressor of NF-kB nuclear factor [84]. Furthermore, rs11556218 in the *IL16* and rs34708521 in the *SPEF2* genes were both associated with thrombosis and pancreatitis related to asparaginase use. The *IL16* gene codes for interleukin-16, a cytokine with known roles in cancer development and inflammatory and autoimmune responses [85]. The *SPEF2* (Sperm Flagellar 2) gene codes for a protein that is required for correct axoneme development, influencing protein dimerization activity [86]. A concept that synergistic interactions between the genetic variants identified in this study is related to asparagine-related toxicities (rs3809849 *MYBBP1A*, rs11556218 *IL16*, and rs34708521 *SPEF2* genes) was proposed [82].

An unbiased transcriptome-wide RNA targeted sequencing discovered that ALL patient leukemic cells with relatively high levels of opioid receptor μ1 (OPRM1) are more sensitive to asparaginase treatment compared to OPRM1-depleted leukemic cells [87]. Stimulation of the opioid receptor leads to the activation of inhibitory Gi-proteins that influence cAMP levels and subsequently induces apoptosis by caspase activation in leukemia cells [88]. It is proposed that OPRM1 can be targeted for effective treatment of asparaginase-resistant ALL patients.

Using a GWAS approach, a single-genetic variant rs738409 in *PNPLA3*, which was strongly associated with hepatotoxicity after induction therapy in pediatric ALL patients, was identified [89]. Patatin-like Phospholipase Domain Containing Protein 3 (PNPLA3 or adiponutrin) is an enzyme involved in triacylglycerol metabolism and signaling [90] and the genetic variant identified in this study leads to the increase of hepatic triglycerides and the induction of fatty liver, thus conferring an increased risk of hepatotoxicity. This finding was confirmed in a mice model in the same study. The validation study confirmed the association of rs738409 in *PNPLA3* with hepatotoxicity during the induction phase of pediatric ALL therapy [75].

In another GWA study, a germline genetic variant, rs4958351, in the *GRIA1* gene, associated with an asparaginase allergy in pediatric ALL patients, was identified [91]. This genetic locus was previously associated with asthma and atopy [92] and the findings strongly support the hypothesis that an asparaginase allergy and asthma share a range of genes that might cause adverse reactions. *GRIA1* encodes a subunit of the AMPA (α-amino-3-hydroxyl-5-methyl-4-isoxazole-propionate) receptor, an ion channel that transmits glutamatergic signals in the brain. The same variant was found to influence some neurologic disorders [93].

The correlation between the *GRIA1* variant, rs4958351, and *E. coli* asparaginase hypersensitivity was confirmed in different childhood ALL subsets [94]. Namely, carriers of at least one A allele at rs4958351 and the T-ALL subtype were at a deceased risk for asparaginase-related hypersensitivity in comparison to the GG genotype. Patients with B-ALL subtypes and the same alleles were at a higher hypersensitivity risk. Interestingly, a lower frequency of asparaginase hypersensitivity was detected among ALL patients with Down syndrome. Moreover, the association between the *GRIA1* variant, rs4958351, and *E. coli* asparaginase hypersensitivity was confirmed in 146 Slovenian pediatric ALL patients [95]. The same association of rs4958351 in the *GRIA1* gene with an asparaginase allergy in pediatric ALL patients was confirmed in another GWAS study, with the additional observation that the risk of allergy was higher in patients treated with native *E. coli* asparaginase than in patients treated with PEGylated *E. coli* asparaginase [77].

A microarray study identified an association of the HLA-DRB1*07:01 allele with asparaginase hypersensitivity and with anti-asparaginase antibodies [96]. Furthermore, HLA-DRB1*07:01 was predicted to have high-affinity binding for asparaginase epitopes. A mechanism was proposed of how an allergy could develop, suggesting that inherited *HLA*-*DRB1* variant alleles produce amino acid variations of the protein whose interaction with asparaginase epitopes is aberrant, leading to a higher frequency of asparaginase hypersensitivity [96].

Also, the HLA-DRB1*07:01 variant allele was confirmed to be associated with asparaginase hypersensitivity using an exome array approach [96]. Moreover, the association of HLA-DRB1*07:01 and asparaginase hypersensitivity, identified in European ALL pediatric patients [96], was confirmed in non-European ALL patients [77].

In the study using next-generation sequencing, it was found that HLA-DRB1*07:01 and *HLA*-DQB1*02:02 alleles were associated with an increased risk of the development of asparaginase hypersensitivity [97]. Furthermore, the HLA-DRB1*07:01-DQA1*02:01-DQB1*02:02 haplotype carriers were positively and significantly associated with an increased risk to asparaginase hypersensitivity. The findings from the previous study were confirmed [96], but after haplotype reconstruction, only the *HLA*-DRB1*07:01-DQB1*02:02 haplotype was associated with asparaginase hypersensitivity [97].

In the study using a genome wide approach, an association of the intronic rs6021191 variant in the *NFATC2* gene with a higher risk of asparaginase hypersensitivity in pediatric ALL patients was found [77]. The presence of the same variant was correlated with higher *NFATC2* mRNA expression [77]. The *NFATC2* gene codes for a cytoplasmic component of the nuclear factor of the activated T cells (NFAT) transcription factor family [98], but its role in asparaginase hypersensitivity is still unclear. It is known that NFATC2 could affect the development and function of regulatory T cells, thus influencing the immune response [99]. Furthermore, a strong association was identified for rs62228256 *NFATC2* and asparaginase-associated pancreatitis [100].

A very recent large GWAS study found and validated variants in the *PRSS1-PRSS2* locus (rs4726576; rs10273639) to be associated with the risk of asparaginase-associated pancreatitis in children with ALL [100]. The pathogenesis of aparaginase-associated pancreatitis in ALL children is the same as in non-asparaginase associated pancreatitis, developed because of alcohol or hyperlipidemia exposure. It is a consequence of the activation of trypsin within pancreatic acinar cells [100].

The non-coding *CNOT3* variant, rs73062673, was confirmed to be strongly associated with a PEG-asparaginase allergy in ALL children in the GWAS [101]. This is the first study taking into account asparaginase enzyme activity measurements to identify asparaginase hypersensitivity. It has been shown that *CNOT3* influences the transcription of MHC class II genes [102]. Moreover, the study pointed out two more genetic variants related to *HLA-DQA1* rs9272131, previously indicted to be involved in allergies, together with variants in the *TAP2* gene, located in close proximity to the *HLA*-*DQA1* variant, also with previously reported connections with asthma and allergy [103]. The association between the *HLA* region and asparaginase hypersensitivity has been described previously [77,96,97], but the potential contribution of an *HLA*-regulating gene is novel.

## 6. Anthracyclines

Anthracyclines (doxorubicin, daunorubicin, epirubicin, and idarubicin) are used to treat a wide range of cancers, including childhood ALL. Daunorubicin and doxorubicin (DOX) are isolated from a natural soil-dwelling bacterium, *Streptomyces peucetius* var. caesius, and from a mutated strain of the same bacterium, respectively [104]. Anthracyclines exert their action through a number of different mechanisms. They inhibit topoisomerase 2-α (TOP2A), which cause double stranded DNA breaks and relax DNA supercoiling during processes of DNA replication and transcription. Anthracyclines interfere with TOP2A dissociation from DNA after making a DNA brake and stop re-ligation [105]. Anthracyclines also intercalate with DNA directly, thus inhibiting biosynthesis of macromolecules, inducing the formation of free radicals and DNA damages and lipid peroxidation, and affecting DNA-binding and alkylation and DNA cross-linking. These combined effects eventually lead to programmed cell death [106].

The benefit of anthracyclines’ use in complex treatment protocols is compromised by cumulative dose-dependent cardiotoxicity [107]. Acute anthracycline-induced cardiotoxicity happens often immediately after the first dose, but delayed chronic anthracycline-induced cardiotoxicity could be presented within one year, a few years, or even decades after the first anthracycline dose.

A number of candidate gene studies brought encouraging results about genes and genetic variants involved in anthracycline-related cardiotoxicity, such as *ABCC1*, *ABCC2*, *ABCC5*, *ABCB1*, *ABCB4*, *CBR3*, *RAC2*, *NCF4*, *CYBA*, *GSTP1*, *CAT*, *SULT2B1*, *POR*, *HAS3*, *SLC22A7*, *SLC22A17*, *HFE*, and *NOS3* [108]. However, large scale studies have pointed out few genetic markers that need to be validated in different cohorts of patients and also in various populations.

A microarray study showed that a synonymous coding variant (L461L) in the *SLC28A3* gene (or human concentrative nucleoside transporter (*hCNT3*)) was highly associated with anthracycline-induced cardiotoxicity [109]. Previous investigations on this nucleoside transporter provided evidence that supports a functional role of this genetic variant in anthracycline-induced cardiotoxicity [110]. The effect of variant rs7853758 in the *SLC28A3* gene on anthracyclines’ transport into cells could be specific for doxorubicin and danorubicin [109]. Besides, it was found that the anthracycline-induced cardiotoxicity was associated with other variants in genes coding for proteins involved in processes known to affect anthracycline ADME, such as *SLC28A1, SLC10A2*, and several ATP–binding cassette transporters (*ABCB1*, *ABCB4*, and *ABCC1*) [109]. This study did not confirm previously determined associations of anthracycline-induced cardiotoxicity with the variants, *ABCC2* rs8187694, *CYBA* rs4673, *RAC2* rs13058338, and *NCF4* rs1883112 [111,112], or variant *CBR3* rs1056892 [108]. However, these associations could be different for adult and childhood patients. Additionally, an analysis conducted in a childhood ALL anthracycline-treated cohort of patients did not confirm the previously detected association of antacycline-induced cardiotoxicity with genetic variants in the catalase gene [113].

Another study has confirmed the association of the variants, rs17863783 in the *UGT1A6* (UGT1A6*4 allele) gene and rs885004 in the *SLC28A3* gene, with anthracycline-induced cardiotoxicity [114]. The *SLC28A3* rs7853758 variant has been associated with a reduced risk of anthracycline-induced cardiotoxicity, i.e., it has a protective role. Furthermore, an effect of rs17583889 and rs17645700 in the histamine N-methyltransferase gene (*HNMT*) was noticed only in children younger than 5 years. Also, the effect of *SULT2B1* rs10426377 was observed in males only. A variant in *ABCB4* (rs4148808) in the promoter region was shown to have an impact on anthracycline-induced cardiotoxicity only in females [114]. These findings need further validation in independent studies.

Further, the same research group revealed two novel variants, rs4982753 in the *SLC22A17* gene and rs4149178 in the *SLC22A7* gene, as predictive markers of anthracycline-induced cardiotoxicity [115]. SLC22A17 (OCT2), an organic cation transporter, is expressed in a variety of tissues, including the heart. It transports naturally occurring nucleosides and nucleotides and several nucleoside-based drugs and, interestingly, shows substrate overlap with concentrative nucleoside transporters, such as SLC28A3, previously related to anthracycline-induced cardiotoxicity [109]. Additional evidence for the association of variants in *SULT2B1* rs10426628 and several antioxidant genes (*CYP2J2, GSTA2, GSTM3, GPX3, SOD2*, and *ABCC9*) was found in this study.

A GWAS using a three-stage genetic association study combined with biological functional analyses identified a nonsynonymous variant in *RARG* (rs2229774, p.Ser427Leu) as being highly associated with anthracycline-induced cardiotoxicity [116]. *RARG* expression has been reported to be particularly high in the heart [117]. RARG has been shown to bind to the *Top2b* promoter [118] and the presence of the rs2229774 variant represses the expression of *Top2b*, finally leading to an anthracycline-induced cardiotoxicity phenotype.

A two-stage study revealed the variant, rs2232228, in the hyaluronan synthase 3 (*HAS3*) gene with a modifying effect on the anthracycline dose-dependent cardiomyopathy risk [119]. Patients who are carriers of the rs2232228 GG genotype did not have any dose-dependent increase of anthracycline-induced cardiomyopathies. However, carriers of the rs2232228 AA genotype were at an increased risk of developing cardiomyopathies when the anthracycline dose was increased. The *HAS3* gene codes for the low-molecular-weight hyaluronan enzyme (HA), an important component of the extracellular matrix, involved in injury processes. Anthracyclines induce apoptosis in heart muscle and injure the cardiomyocytes. Cardiac fibroblasts repair and remodel the heart using the extracellular matrix with accumulated HA as a scaffold [120].

## 7. Thiopurine Drugs

6-Mercaptopurine and 6-thioguanine are thiopurine drugs used in the treatment of childhood ALL. These drugs are purine analogs, which are metabolically transformed to thioguanine nucleotides (TGN) capable of becoming incorporated into DNA, which leads to cell death.

The thiopurine S-methyltransferase (TPMT) is an enzyme that detoxifies thiopurine drugs by methylation of thiopurine analogs, which interferes with their incorporation into DNA. Patients’ TPMT activity depends on variants in the *TPMT* gene and this trait is codominantly inherited: Patients who carry one non-functional allele have intermediate TPMT activity, while patients with two non-functional alleles have very low TPMT activity [121]. Three common variants of the *TPMT* gene (rs1800462, rs1800460, and rs1142345) account for most cases of inherited TPMT deficiency, and their distribution is population specific. In Caucasians and Africans, there is a higher prevalence of non-functional alleles in comparison to East Asian populations. Also, in Caucasians, the most frequent non-functional allele is the *3A (consisting of both rs1800460 and rs1142345 minor variants) allele, while in East Asians, the *3C (rs1142345) allele is the most frequent. Thiopurine dosage and toxicity have been repeatedly and consistently associated with TPMT activity and genetics irrespective of ethnicity or underlying disease. *TPMT* and thiopurines represent one of the first and best documented gene–drug pairs in pharmacogenomics and this knowledge is used for the benefit of patients through therapy individualization [122].

The TPMT enzyme requires S-adenosylmethionine for its activity and this cofactor contributes to TPMT enzyme stability. Intracellular S-adenosylmethionine levels depend on the folate cycle, especially on the activity of the methylenetetrahydrofolate reductase (MTHFR) enzyme. Using a candidate gene approach, TPMT activity and thiopurine toxicity were associated with genetic variants important for the folate cycle [123,124]. To elucidate the genetics of TPMT activity, two large GWAS studies analyzed liver and erythrocyte TPMT enzyme activity in childhood ALL patients and healthy controls. The results showed that TPMT enzyme activity was associated only with variants in the *TPMT* gene, which underlined the utility of *TPMT* genotyping in clinical settings [125,126], but also undermined the role of folate cycle genes for TPMT activity and thiopurine clearance. 

Before agnostic approaches using GWAS were available, other candidate gene variants were tested for their associations with thiopurine toxicity. Pharmacogenes that encode transporters and enzymes involved in the clearance of thiopurine drugs have been in the focus, in particular *ITPA* and *ABCC4*. The *ITPA* enzyme catalyzes hydrolysis of the pyrophosphate group from purine analogs triphosphates, which interferes with their incorporation into DNA [127], while the *ABCC4* transporter exports thiopurine drugs and their metabolites [128]. Lower activity variants of *ITPA* and *ABCC4* [129] genes have been associated with a diminished tolerance of thiopurine therapy, however, this is inconsistent [130,131].

A candidate gene approach could not explain all the toxicity of thiopurine drugs, especially in East-Asian patients. Despite having a smaller burden of *TPMT* no-function alleles, East Asians have a lower tolerance of thiopurine drugs compared to other populations [132]. ITPA and ABCC4 deficiencies are more prevalent in East Asians, which served as an explanation for the lower 6-MP tolerance in this population. However, a GWAS involving two large cohorts of childhood ALL patients introduced a new pharmacogene as a major determinant of 6-MP intolerance, which is particularly relevant for East Asians [132]. Variant rs116855232 of the *NUDT15* gene showed both a strong association and clinical importance. Patients with TT and CT genotypes could tolerate only around 10% and 75% of the dose tolerated by patients with the CC genotype. Besides *NUDT15*, the only pharmacogene associated with 6-MP intolerability found by the GWAS study was *TPMT*, which questioned the clinical importance of other pharmacogenes involved in 6-MP clearance. The importance of NUDT15 for thiopurine inactivation and cytotoxicity was subsequently shown both in vitro and in vivo [133]. Also, the association of non-functional *NUDT15* alleles with 6-MP intolerance was corroborated in multiple studies [131,133,134,135]. Based on overwhelming evidences that emerged in the last 5 years, *NUDT15* testing is now recommended prior to the onset of thiopurine therapy [136].

Protein kinase C and casein kinase substrate in neurons’ protein 2 (PACSIN2) was brought into the focus of pharmacogenomics of thiopurine drugs after a GWAS study involving cell lines in which variant rs2413739 showed the highest association with TPMT activity [137]. This result was subsequently corroborated, but only for ALL patients, while in inflammatory bowel disease (IBD) patients and healthy subjects, TPMT activity was not associated with the *PASCIN2* rs2413739 variant [138]. However, the association of *PASCIN2* variants with TPMT enzyme activity was not shown either for ALL patients or for healthy controls in GWAS studies [125,126]. Several studies also dealt with the association of the *PASCIN2* variant with thiopurine toxicity in ALL patients and reported that rs2413739 is a factor of thiopurine-related toxicity [137,138,139]. Although there are only a few studies on the association of *PACSIN2* gene variants with thiopurine therapy in ALL patients, they included a considerable number of patients and came to similar conclusions. As for non-ALL patients, it was shown that the T allele of the *PACSIN2* rs2413739 variant was not associated with a higher toxicity, although the study was sufficiently powered [140]. Further analyses, optimally including both functional and association studies, are needed to determine whether *PACSIN2* is a factor related to thiopurine intolerance. 

Another genetic determinant of thiopurine therapy came into focus after two GWAS studies concurrently and independently associated somatic mutations in the 5′-Nucleotidase, Cytosolic II (*NT5C2*) gene with relapse in childhood ALL patients [141,142]. These mutations were also associated with early rather that late relapse, which underlines their deleterious effect for disease progression. Subsequent analyses showed that acquired mutations related to relapse activate NT5C2, an enzyme that dephosphorylates thiopurine nucleotide monophosphates, making them inactive and prone to export from the cell [141,142]. In a latter study, in which deep sequencing of cancer associated genes was carried out, *NT5C2* mutations were also identified among relapse-associated, somatic mutations [143], underlining the importance of *NT5C2* mutations as prognostic biomarkers related to thiopurine therapy in ALL patients.

A candidate transcript approach can also direct the analysis of pharmacogenes’ expression signatures to enhance the prediction of drug toxicity and response. For instance, it was shown that the quantity of *TPMT* transcript can be modulated by variants in the gene promoter, particularly a variable number of tandem repeats (VNTR) [144,145]. However, the impact of the *TPMT* expression profile on thiopurine effects in pediatric ALL patients is scarcely investigated. In one study, it was shown that the *TPMT* expression level was significantly higher during the maintenance phase of therapy than on diagnosis, being the highest in the early stage of the maintenance phase. Also, carriers of specific VNTRs differ in *TPMT* expression levels, which could be important to consider before the onset of maintenance therapy [146].

Many microarray studies have been focused on the expression signatures of ALL relapse [147,148,149,150,151], however, studies dealing specifically with transcriptome of thiopurine or methotrexate in vivo resistance are limited. Zaza and colleagues investigated the correlation of gene expression profiles with the level of thioguanine nucleotides in pediatric ALL patients at diagnosis after initial treatment with 6-MP alone or the combination of 6-MP and MTX. The study identified 60 genes (31 positively and 29 negatively correlated) in 6-MP and 75 genes (50 positively and 25 negatively correlated) in 6-MP + MTX treatment that were significantly associated with TGN accumulation. These two sets of genes did not overlap, indicating different pathways involved in these two therapeutic approaches as well as the fact that the effects of combination therapy are not additive. In the 6-MP treatment, the most associated genes with TGN levels were xanthine oxidase (*XDH*), solute carrier family 29 member 1 (*SLC29A1*), adenosine deaminase (*ADA*), and other genes related to cell proliferation and apoptosis (*CASP7, TOPBP1, ANAPC5, CCT4*) [152]. Xanthine oxidase is, besides the TPMT enzyme, involved in the inactivation of 6-MP and it is the main target of allopurinol, which is used in combination with azathioprine to increase the shunting of 6-MP down the pathway of producing active metabolites. However, compared to TPMT, little attention has been given to this oxidation pathway [153]. The SLC29A1 influx transporter has been positively correlated with the cytotoxicity of nucleoside analogs in human cancer cell lines [154]. In addition to previous results, Zaza and colleagues demonstrated that the inhibition of the SLC29A1 transporter led to approximately a 40% reduction of thioguanines [152]. Contrary to 6-MP alone, the 6-MP + MTX combination yielded genes involved in adenosine triphosphate synthesis, such as *SLC25A3*, *ATP50*, *COX5B*, and *COX7A2L*, and other genes implicated in protein synthesis (*RPS19, RPL18, RPS25, RPL23*) and translation factors (*EEF1G*, *EIF3S5*, *eIF3k*) [152].

The study of Hogan and colleagues, which examined pediatric B-ALL patients’ paired samples taken at diagnosis and relapse, showed that relapse timing (early or late) was associated with distinct gene expression signatures. Particularly, late relapse was associated with the up-regulation of genes involved in nucleotide biosynthesis and folate metabolism, such as *PAICS*, *TYMS*, *CAD*, *ATIC*, and *GART*, which is interesting given that 6-MP and MTX are crucial for maintenance therapy [149]. Also, somatic deletion and consequently decreased expression of the *MSH6* gene, which is involved in the mismatch repair mechanism and was previously associated with thiopurine resistance, has also been detected at the time of relapse [149,151].

Besides identifying pharmacogenes involved in drug response, pharmacotranscriptomic data can be useful in designing predictive algorithms of patient clinical outcomes. Beesley and colleagues used gene expression data of 15 T-ALL cell lines and their sensitivity to 10 therapeutics commonly used in ALL treatment to generate a model which could predict in vivo resistance and therefore patient outcomes. The designed model was validated on the microarray data of three independent pediatric T-ALL cohorts, showing the clinical relevance of identified drug–gene signatures. Moreover, it has been demonstrated that the expression signatures most useful for the accurate prediction of relapse were associated to 6-MP resistance. Genes whose expression was associated to thiopurine resistance were mostly involved in biological pathways, such as gene expression, differentiation/development, cell growth and proliferation, and cell death. Among these, novel associations with the thiopurine drug response were observed for sulfite oxidase (*SUOX*) and multidrug resistance protein (*ABCC1* or *MRP1*) genes. Interestingly, *SUOX* belong to the same family of oxotransferases as xanthine oxidase (XO) and the activity of both relies on molybdenum metabolites from a common biosynthetic pathway. Thus, it is possible that the altered expression of *SUOX* could influence XO activity by modifying the level of molybdenum metabolites and indirectly thiopurines’ detoxification [155].

Here, we find a good example showing how a wide transcriptome approach is more useful than a candidate transcript approach in identifying novel potential expression biomarkers of a poor drug response. Interestingly, no significant association was found for *TPMT* gene expression in either of the aforementioned microarray studies.

## 8. Methotrexate

Methotrexate is one of the key drugs of ALL treatment, which is given in all phases across different ALL therapy protocols, either systemically or locally. Methotrexate enters cells primarily via the solute carrier family 19 member 1 (SLC19A1) transporter, followed by its polyglutamination catalyzed by the folylpolyglutamate synthase (FPGS) enzyme, which further activates the drug and hinders its clearance. MTX and MTX polyglutamates (MTX-PG) inhibit dihydrofolate reductase (DHFR), a key enzyme in the folate cycle essential for the replenishment of active folate forms used in nucleotide synthesis and methylation reactions. MTX-PG also inhibits the thymidylate synthase (TYMS) enzyme, necessary for the synthesis of tymidine nucleotides. Another important enzyme indirectly impacted by MTX is MTHFR, an enzyme which facilitates the synthesis of 5-methyltetrahidrofolate, ultimately used for the methylation reaction at the expense of 5,10 methylentetrahydropholate, necessary for thymidylate synthesis. As a consequence of MTX therapy, important cellular processes, including DNA synthesis and methylation, are tempered, which contributes to MTX anti-cancer effects and MTX-related toxicity.

Response to MTX therapy is associated with the activity of key enzymes and transporters involved in the MTX and folate metabolic pathway. For instance, DHFR upregulation is associated with poor survival of childhood ALL patients [156], while higher intracellular levels of long-chain MTX-PG, correlated with higher FPGS activity, is a factor of improved survival of ALL patients [157]. The complex folate and MTX metabolic pathway allowed for the selection of several candidate pharmacogenes that encode enzymes and transporters involved in MTX anti-cancer effects and clearance. MTX-associated pharmacogenes contain numerous genetic variants that are frequent in human populations and are coupled with functional consequences for the activity of corresponding proteins. For example, one of the most extensively studied variants in the pharmacogenetics of MTX is a common rs1801133 (677C > T) variant in the *MTHFR* gene, which causes amino acid substitution and decreased protein activity. Though several studies conducted on childhood ALL patients showed that the T allele of rs1801133 is associated with toxicity [129,158,159,160], a few studies showed no association [161,162] or even a protective effect [163]. Two recent meta-analyses tried to settle this dilemma, but they reached opposite conclusions [164,165]. A number of similar studies relying on the candidate gene approach have been carried out in order to find genetic markers of MTX related toxicity and therapy response (recently reviewed by Giletti and colleagues [166]). However, despite extensive analysis of multiple candidate genes, none of the genetic markers so far have been used in MTX therapy protocols due to the lack of a clear association with the response and/or toxicity.

One of the most promising pharmacogenes previously not analyzed in the context of MTX pharmacokinetics, *SLC1O1B1*, has emerged following a large GWAS study conducted by Trevino and colleagues in 2009. The results showed that only *SLCO1B1* variants (intronic rs11045879 and rs4149081 tied to functional rs4149056) are associated with MTX clearance and GI toxicity and this result was replicated in an independent cohort [167]. An even larger replication GWAS study, enrolling around 1300 childhood ALL patients, reported essentially the same conclusions reached by Trevino and colleagues [168], associating rs4149081, rs11045879, rs11045821, and functional rs4149056 with MTX clearance. The contribution of functional variants in the *SLCO1B1* gene to MTX clearance variability is around 10% [169]. An association of *SLCO1B1* variants with MTX clearance and toxicity was corroborated using the candidate gene approach [170,171,172]. *SLCO1B1* variants were also associated with the event-free survival of ALL patients [173], however, another study did not find an association between functional rs4149056 and the risk of relapse [172].

In search of pharmacogenomic markers of MTX therapy, a study by Lopez-Lopez enrolled 151 pediatric B-ALL patients to analyze more than 300 variants in 12 candidate transporter genes related with MTX transport. The results showed that only variants in the *ABCC2* and *ABCC4* gene are in relation with MTX plasma levels [174], which is a surrogate marker of MTX-related toxicity [170]. Variants in the *SLCO1B1* gene did not reach a significant level of association after the correction for multiple testing [174]. None of the two GWAS studies, nor a study focused on MTX transporters, showed significant associations (after the correction for multiple testing) between MTX clearance or MTX plasma levels and variants in the *SLC19A1* gene [167,168,174], by far the most studied MTX transporter encoding pharmacogenes.

Alternations in the expression of candidate genes involved in MTX transport (*SLC19A1*, *ABCC1-4*, *ABCG2*), MTX metabolism (*FPGS*, *FPGH*), as well as MTX target enzyme genes (*DHFR*, *TYMS*) were investigated in relation to MTX resistance [175,176,177]. Although low *SLC19A1* and *FPGS* as well as high *DHFR* and *TYMS* expression levels have been correlated with poor patient MTX response [157,178,179,180,181], some important factors, such as genomic landscape, MTX dosage (high or low), and the subtype of ALL, should be taken into account. Particularly, it was shown that precursor B-cell ALL patients display a higher MTX sensitivity than T-cell ALL patients [157,182,183]. In line with this are results showing higher *FPGS* mRNA expression as well as FPGS enzyme activity in B-cells ALL, and higher levels of *DHFR* and *TYMS* mRNA in T-cells ALL [157,180]. Moreover, children with hyperdiploid ALL (more than 50 chromosomes) showed increased MTX sensitivity, measured by increased MTX-PG accumulation, which was associated with higher *SLC19A1* expression as a result of extra copies of chromosome 21, where the gene is located. However, this effect was seen only if patients were treated with low doses of MTX, during which the main mechanism of the antifolates’ entry is via the SLC19A1 [178,184]. In contrast, patients carrying the *E2A-PBX1* and *TEL-AML1* gene fusions displayed a decreased MTX-PG accumulation associated with the diminished expression of *SLC19A1* in *E2A-PBX1* and the elevated expression of *ABCG2* in *TEL-AML1* ALL patients [184]. Additionally, Kager and colleagues established distinct in vivo folate pathway gene expression patterns, which provided an 83% accuracy for correctly assigning the ALL genetic subtype or lineage. These results point out the importance of ALL subtype–specific strategies to overcome MTX drug resistance.

Using a transcriptome wide approach, Sorich and colleagues gave insight into the gene expression signatures of good and poor MTX in vivo responses in de novo pediatric ALL patients, identifying 50 genes (21 positively and 29 negatively) associated specifically with the MTX antileukemic effect [185]. These genes included the ones involved in nucleotide metabolism (*TYMS* and *CTPS*), cell proliferation and apoptosis (*BCL3*, *CDC20*, *CENPF*, and *FAIM3*), and DNA replication or repair (*POLD3, RPA3, RNASEH2A, RPM1*, and *H2AFX*). The study also showed that a low expression of the *DHFR*, *TYMS*, and *CTPS* genes was associated with poor therapeutic response and prognosis A lower expression of these genes is associated with decreased processes of DNA synthesis and cell proliferation, making cells less susceptible to the effects of MTX drugs [185]. As indicated, this is not in contrast to previous results showing that a higher expression of *TYMS* and *DHFR*, due to promoter variants, leads to a worse prognosis, which could have an effect once remission is achieved [156,186,187]. In the microarray study of Kager and colleagues, a significant correlation was demonstrated between *TYMS*, *MTHFD1*, and *RUVBL2* expression and MTXPG accumulation in B-ALL not carrying cytogenetic abnormalities whereas *MTHFD2*, *PPAT*, and *RUVBL2* expression was associated with MTXPG accumulation within T-ALL. A significant correlation between *ABCG2*, *ABCC4*, and *TYMS* expression and the cytotoxic effects of MTX in ALL B-cells not carrying cytogenetic abnormalities has also been found [184].

A list of the pharmacogenomic and pharmacotranscriptomic markers of the drug response or toxicity discovered or validated using high-throughput technologies is summarized in Table 1.

## 9. Conclusions

Pharmacogenomics and pharmacotranscriptomics in childhood ALL are the focus of numerous studies due to the availability of high quality data for the assessment of an association between the genomic and transcriptomic profiles of patients and their response to therapy. Valuable data are accessible because of standardized similar protocols that are used for the treatment of pediatric ALL in many populations, with a similar efficacy and side effects in all of them.

Recent reviews have summarized data related to pharmacogenomics and pharmacotranscriptomics in childhood ALL [188]. In this review, we paid attention to the studies in which high-throughput technologies were used. 

We found several reports of studies in the field of pharmacogenomics in childhood ALL using high-throughput technology. In most of them, microarray technology was used, while the reports in which targeted DNA sequencing, WES, or WGS was applied were significantly fewer. It is probably due to the fact that arrays were more cost-effective than sequencing technology. However, at this moment, sequencing technologies are more effective and less expensive and the shift towards sequencing-based studies in this field is obvious. A very small number of studies of somatic mutations relevant for pharmacogenomics in ALL have been conducted. Since a high coverage is required for the detection of somatic mutations in the samples with a large number of subclones, characteristic for leukemia, deep targeted sequencing is indispensable for that type of analysis. Future research should be directed towards association studies of somatic mutations and drug resistance in pediatric ALL.

An even smaller number of studies on pharmacotranscriptomic markers have been performed. RNA seq methodology is used only in a few studies. Studies of regulatory RNA (microRNA of long non-coding RNA) are deficient. 

Studies based on high-throughput analyses led to many beneficial discoveries. Since numerous pharmacogenomic/pharmacotranscriptomic studies have found differences in the prevalence of clinically significant variants between populations, propositions of creating databases concerning the pharmacogenetic/pharmacotranscriptomic markers in different populations have sprung up. One of these databases is FINDbase [189], a comprehensive database containing population frequency data of clinically relevant variants. In another pharmacogenomic study [190], significant interpopulation differences have been reported in seven European population concerning seven important pharmacogenomic biomarkers, which change the drug efficacy and/or toxicity of up to 51 treatment modalities. These results could be beneficial in creating accurate population-based preemptive pharmacogenomic testing.

Hypothesis driven studies using a candidate gene approach might be an inefficient way to discover novel pharmacogenomic markers because candidate genes outside the well-studied network of drug absorption, distribution, metabolism, and excretion (ADME) or drug targets are often missed. Besides, the effect of investigated genetic variants is often not clear and functional analyses are often scarce or contradictory. For instance, the effect of the nonsynonimous variant, rs1051266 (*SLC19A1* 80G > A), on MTX transport efficacy is ambiguous, as it is suggested that the minor A allele has both a higher and lower affinity to MTX, as well as having a marginal functional effect. Nevertheless, a number of candidate gene studies have tried to relate this variant with MTX levels and toxicity, but the results were also inconclusive. Moreover, hypothesis free, high-throughput analyses could not confirm the association of MTX pharmacokinetics and the rs1051266 variant, even though the minor allele frequency is almost 50%. Instead, GWAS studies introduced a novel, SLC19A1 pharmacogene as a factor of MTX, whose significance was later confirmed.

Although high-throughput technology has brought a significant increase of knowledge in pharmacogenomics and pharmacotranscriptomics in childhood ALL, most GWAS/TWAS studies have provided contradictory results. There could be several reasons for this. First, patient cohorts selected for the GWAS/TWAS are usually not uniform. The genetic profile of pediatric ALL is complex and heterogenous, which has led to the treatment of patients according to the stratification principle. A lack of knowledge of the complete biomarker profile at the beginning of the disease could lead to false conclusions at the end. Additionally, the phenotype endpoints should be precisely defined. The time of molecular-genetics analysis is critical and should always be performed in the same phase of the same treatment protocol, especially when expression studies interpret pharmacotranscritomics markers. It is also particularly hard to determine the grade of drug side effects when small children are reporting, for example, the neuropathy pain level. Furthermore, given the complexity of GWAS/TWAS, multiple sources of false positive and false negative errors exist. The inconsistencies in the GWAS/TWAS results are caused either by the design of experiment itself or by the genotype/transcriptome calling process [191].

Many authors consider small sample sizes as being responsible for limited outcomes in pharmacogenomic/pharmacotranscriptomic studies in pediatric ALL. However, some of the studies have included a significant number of patients [69,77,96].

Big data generated in candidate gene/transcripts studies have been enormously expanded due to the implementation of high-throughput technologies. In the last two decades, an accumulation of data in the field of pharmacogenetics/pharmacogenomics has been achieved, reaching more than 20,000 new citations in PubMed. More than 3500 associations between pharmacogenes and pharmacogenomic variants and the efficacy/toxicity of drugs have been validated and can be considered to have strong evidence. More than 200 drugs have drug labels containing information of the mandatory or recommended preemptive pharmacogenomic testing (https://www.fda.gov/Drugs/ScienceResearch/ucm572698.htm).

**Figure 1:**
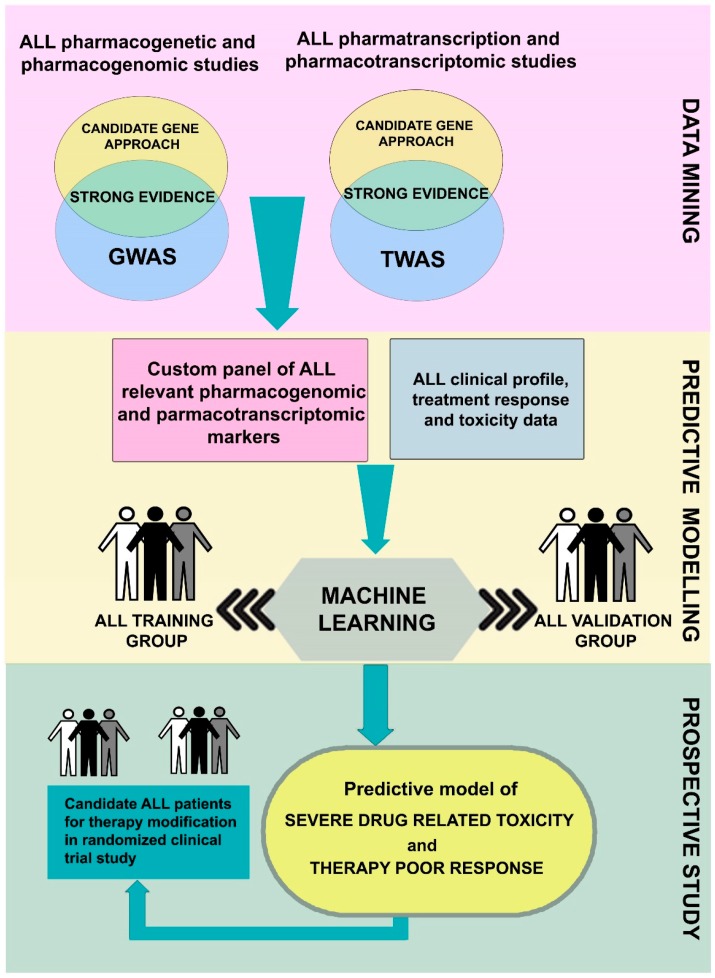
Diagram of the steps in designing a predictive model of childhood acute lymphoblastic leukemia (ALL) patients’ drug related toxicity and outcomes using pharmacogenomic and pharmacotranscriptomic data. Data mining of the current literature and the selection of biomarkers that showed strong evidence for an association with the treatment response and toxicity can be used for the creation of a custom panel for genomic and transcriptomic profiling. Along with patients’ clinical data, molecular data obtained via pharmacogenomic and pharmacotranscriptomic profiling could be utilized for the design of a prediction model using machine learning algorithms. This form of artificial intelligence requires a training group of pediatric ALL patients to learn how selected molecular markers relate to each other to predict specific outcomes, such as patients’ drug responses. Also, validation of the model is needed on an independent ALL cohort to test the model’s performance. If the model could predict patients at risk of severe drug related toxicity or poor response with sufficient accuracy, protocol modifications for these patients might be attempted using a randomized clinical trials approach.

However, it is necessary to use the available big data in translational research to obtain data that is usable in clinical practice. For that reason, research efforts must be focused on the development of data analysis. Data mining of the current literature and the selection of biomarkers that showed strong evidence for an association with the treatment response and toxicity can be used for the creation of a custom panel for genomic and transcriptomic profiling. Along with patients’ clinical data, molecular data obtained via genomic and transcriptomic profiling could be utilized for the design of a prediction model using machine learning algorithms. This form of artificial intelligence requires a training group of ALL patients to learn how selected molecular markers relate to each other to predict specific outcomes, such as patients’ drug responses. Also, validation of the model is needed on an independent pediatric ALL cohort to test the model’s performance. If the model could predict patients at risk of severe drug related toxicity or poor response with sufficient accuracy, protocol modifications for these patients might be attempted using a randomized clinical trials approach (Figure 1).

A true understanding of the processes leading to disease development and mechanisms of treatment efficacy and toxicity, as well as gaining new knowledge from big data obtained in large omics studies and validation studies from various populations, could be the way to achieve the ultimate goal of all biomedical professionals: Bringing real personalized treatment from the bench to the bedside.

The growing knowledge in pharmacogenomics and pharmacotranscriptomics in pediatric ALL, produced by molecular profiling of patients using high-throughput technology, as well as the development of bioinformatics tools and the implementation of artificial intelligence, are expected to improve the treatment of children with ALL through the individualization of therapy for each patient. The door for personalized medicine is wide-open in the clinical practice of pediatric ALL.

## Figures and Tables

**Table 1:** Pharmacogenomic and pharmacotranscriptomic markers of the drug response or toxicity discovered or validated using high-throughput technologies. WGS: whole genome sequencing; WES: whole exome sequencing; DEX: dexamethasone; GC: glucocorticoids; 6-MP: 6-mercaptopurine; MTX: methotrexate; EFS: event free survival; OS: overall survival; *: protective role.

Pharmacogene	Variant or RNA	Effect	Methodology	References
Glucocorticoid drugs				
*ABCB1, WT 1-AS*	rs6007758, rs41488548, rs10264856, rs4728709	Higher clearance of DEX	Microarray	[33]
*SERPINA6*	rs12589136	Higher plasma cortisol levels	WES	[34]
*Intergenic variant*	rs10989692	Increased risk of osteonecrosis	WES	[35]
*ACP-1*	Multiple SNPs	Increased risk of osteonecrosis	Microarray	[36]
*hsa-miR-142-3p, hsa-miR-17-5p*	miRNA	High correlation with GC resistance	Omni-Search	[40]
*EMP1*	mRNA	Higher expression in prednisone poor responders	Microarray	[41]
*CASP1, NLRP3*	mRNA	High expression and subsequent high GC resistance	Microarray	[45]
*SMARCA4, ARID1A, SMARCB1*	mRNA	Decreased expression is associated with GC resistance	Microarray	[46]
*CREBBP*	somatic mutations	Presence of damaging mutations leads to GC resistance	Microarray	[12]
Vincristine				
*CEP72*	rs924607	vincristine-related peripheral neuropathy	Microarray	[58]
*ABCC2*	rs374006 rs12826	vincristine-related peripheral neuropathy	Targeted DNA sequencing	[63]
SYNE2, MRPL47, BAHD1 *	rs2781377 rs10513762 rs3803357 *	vincristine-related peripheral neuropathy	WES	[64]
*COCH*	rs1045644 rs7963521	vincristine-related peripheral neuropathy	Microarray	[69]
*miR-125b, miR-99a, miR-100*	microRNA	resistance to vincristine	microRNA expression study	[72]
*miR-300, DROSHA*	rs12894467 rs639174	vincristine-related peripheral neuropathy, vomits	Microarray	[74]
*miR-3117-3p, miR-4481*	rs12402181 rs7896283	vincristine-related peripheral neuropathy	Microarray	[75]
Asparaginase				
*MYBBP1A, IL16, SPEF2*	rs3809849 rs11556218 rs34708521	allergy, pancreatitis and thrombosis related to asparaginase, EFS, OS	WES	[82]
*OPRM1*	microRNA	resistance to asparaginase	genome-wide RNAi screening	[87]
*PNPLA3*	rs738409	elevated alanine transaminase (ALT) levels leading to hepatotoxicity	Microarray	[89]
*GRIA1*	rs4958351	asparaginase hypersensitivity	Microarray	[91]
*HLA-DRB1*	HLA-DRB1*07:01	asparaginase hypersensitivity	Microarray	[95]
*NFATC2, HLA-DRB1, GRIA1*	rs6021191 HLA-DRB1*07:01 rs4958351	asparaginase hypersensitivity	Microarray	[77]
*HLADRB1, HLADQ1*	HLADRB1*07:01 *HLA*-DQB1*02:02	asparaginase hypersensitivity	targeted DNA sequencing	[97]
*PRSS1-PRSS2 locus* *NFATC2*	rs4726576 rs10273639 rs62228256	asparaginase hypersensitivity, pancreatitis	Microarray	[100]
*CNOT3, HLADQA1, TAP2*	rs73062673 rs9272131	asparaginase hypersensitivity	Microarray	[101]
Anthracyclines				
*SLC28A3*	rs7853758	anthracycline-induced cardiotoxicity	Microarray	[109]
*UGT1A6, SLC28A3*	rs17863783 rs885004 rs7853758 *	anthracycline-induced cardiotoxicity	Microarray	[114]
*SLC22A17, SLC22A7*	rs4982753 rs4149178	anthracycline-induced cardiotoxicity	Microarray	[115]
*RARG*	rs2229774	anthracycline-induced cardiotoxicity	Microarray	[116]
*HAS3*	rs2232228	anthracycline-induced cardiotoxicity	Microarray	[119]
Thiopurine drugs				
*TPMT, NUDT15*	rs1142345, rs116855232	6-MP dose intensity	WGS	[132]
*TPMT*	rs1142345	TPMT activity	WGS	[125,126]
*PACSIN2*	rs2413739, mRNA	TPMT activity	WGS, RNA seq	[137]
*NT5C2*	somatic mutations	Relapse	WES, RNA seq	[141,142]
*XDH, SLC29A1, ADA, CASP7, TOPBP1, ANAPC5, CCT4*	mRNA	Level of TGN after initial MP treatment	Microarray	[152]
*SLC25A3*, *ATP50*, *COX5B*, *COX7A2L*; *RPS19*, *RPL18*, *RPS25*, *RPL23*; *EEF1G, EIF3S5, eIF3k*	mRNA	Level of TGN after initial 6-MP+MTX treatment	Microarray	[152]
*PAICS, TYMS, CAD, ATIC*, *GART, MSH6*	mRNA	Late relapse, probably related to 6-MP and MTX	Microarray	[149]
*SUOX, ABCC1*	mRNA	Thiopurine resistance	Microarray	[155]
Methotrexate				
*SLCO1B1*	rs4149081, rs11045879, rs11045821, rs4149056	MTX clearance	WGS	[167,168]
*ABCC2, ABCC4*	rs3740065, rs9516519	MTX plasma level	Targeted DNA sequencing	[174]
*DHFR, TYMS*, *CTPS; BCL3*, *CDC20*, *CENPF*, *FAIM3; POLD3, RPA3, RNASEH2A, RPM1*, *2AFX*	mRNA	Reduction of circulating leukemia cells after initial treatment	Microarray	[185]
*DHFR, TYMS*	mRNA	5-year disease free survival	Microarray	[185]
*TYMS, MTHFD1, RUVBL2*	mRNA	MTX-PG accumulation after high dose MTX treatment in nonhyperdipoid B-ALL	Microarray	[184]
*MTHFD2, PPAT, RUVBL2*	mRNA	MTX-PG accumulation after high dose MTX treatment in T-ALL	Microarray	[184]
*ABCG2, ABCC4, TYMS*	mRNA	MTX cytotoxic effect in nonhyperdipoid B-ALL, as measured by the reduction of circulating ALL cells	Microarray	[184]
